# Particle Image Velocimetry Algorithm Based on Spike Camera Adaptive Integration

**DOI:** 10.3390/s25206468

**Published:** 2025-10-19

**Authors:** Xiaoqiang Li, Changxu Wu, Yichao Wang, Hongyuan Li, Yuan Li, Tiejun Huang, Yuhao Huang, Pengyu Lv

**Affiliations:** 1 School of Mechanics and Engineering Science, Peking University, Beijing 100871, China; xiaoqiangli@pku.edu.cn (X.L.); 2501111724@stu.pku.edu.cn (Y.W.); lihongyuan@pku.edu.cn (H.L.); 2 School of Advanced Manufacturing and Robotics, Peking University, Beijing 100871, China; 3 Vidar Future Technology (Beijing) Co., Ltd., Beijing 100193, China; huangyh@vidar.cn; 4 School of Artificial Intelligence, Beijing University of Posts and Telecommunications, Beijing 100876, China; 2024141153@bupt.cn; 5 School of Computer Science, Peking University, Beijing 100871, China; yuanli@pku.edu.cn (Y.L.); tjhuang@pku.edu.cn (T.H.)

**Keywords:** particle image velocimetry, high speed camera, neuromorphic vision sensor, spike camera, adaptive integration, overexposure

## Abstract

In particle image velocimetry (PIV), overexposure is particularly common in regions with high illumination. In particular, strong scattering or background reflection at the liquid–gas interface will make the overexposure phenomenon more obvious, resulting in local pixel saturation, which will significantly reduce the particle image quality, and thus reduce the particle recognition rate and the accuracy of velocity field estimation. This study addresses the overexposure challenges in particle image velocimetry applications, mainly to address the challenge that the velocity field cannot be measured due to the difficulty in effectively detecting particles in the exposed area. In order to address the challenge of overexposure, this paper does not use traditional frame-based high-speed cameras, but instead proposes a particle image velocimetry algorithm based on adaptive integral spike camera data using a neuromorphic vision sensor (NVS). Specifically, by performing target-background segmentation on high-frequency digital spike signals, the method suppresses high illumination background regions and thus effectively mitigates overexposure. Then the spike data are further adaptively integrated based on both regional background illumination characteristics and the spike frequency features of particles with varying velocities, resulting in high signal-to-noise ratio (SNR) reconstructed particle images. Flow field computation is subsequently conducted using the reconstructed particle images, with validation through both simulation and experiment. In simulation, in the overexposed area, the average flow velocity estimation error of frame-based cameras is 8.594 times that of spike-based cameras. In the experiments, the spike camera successfully captured continuous high-density particle trajectories, yielding measurable and continuous velocity fields. Experimental results demonstrate that the proposed particle image velocimetry algorithm based on the adaptive integration of the spike camera effectively addresses overexposure challenges caused by high illumination of the liquid–gas interface in flow field measurements.

## 1. Introduction

**Figure 1:**
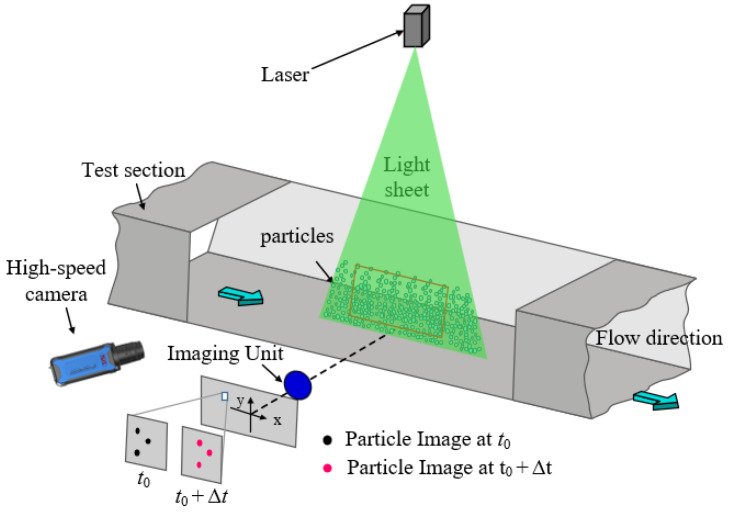
Schematic diagram of particle image velocimetry principle [2].

Particle Image Velocimetry (PIV), as a noncontact and nonintrusive flow field measurement technique, is fundamentally based on tracking particle displacement using cross-correlation algorithms [1]. The PIV system includes lasers to illuminate particles, high-speed cameras for data acquisition, tracer particles to track flow fields, and particle image velocimetry algorithms to estimate flow fields, as shown in Figure 1. This method has extensive applications in research domains such as aerodynamics, microfluidics, biofluids, and combustion studies.

**Figure 2:**
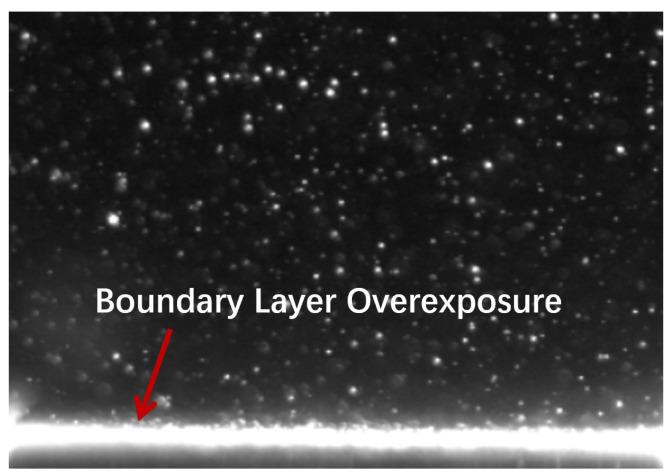
Particle image boundary overexposure diagram [4].

In PIV experiments, the illumination of particle images frequently varies due to factors such as differential particle motion and scattering characteristics, resulting in inconsistent intensities for the same particle across consecutive frames [3]. Overexposure is particularly pronounced near highly reflective or refractive interfaces. When the laser sheet illuminates gas–liquid or solid–liquid boundaries, strong specular reflections and refraction effects can saturate local pixels, causing blooming artifacts. This issue is particularly pronounced in high-gradient or high illumination scenarios, such as the particle image during the water entry of a vehicle, as shown in Figure 2, manifesting itself as:excessive regional illumination dynamic range causes frame-based image sensors to produce overexposed particle images that are unsuitable for the cross-correlation algorithm. Such an overexposure not only obscures the true particle signals but also introduces spurious bright spots, thereby reducing the signal-to-noise ratio and compromising subsequent particle identification and velocity vector estimation.

A key strategy to address the problem of overexposure lies in improving the imaging performance of high-speed cameras. Currently, cameras used for particle image velocimetry can be broadly categorized into two groups: conventional frame-based high-speed cameras and novel neuromorphic vision sensors, each of which will be discussed in detail in the following sections.

### 1.1. Frame-Based High-Speed Cameras

Frame-based high-speed cameras employ high frame rates and short exposure times to capture rapidly moving particles [5], effectively reducing motion blur and saturation. The conventional approach focuses on optimizing the imaging quality of these cameras by adjusting the exposure parameters, illumination intensity, and optical setup to improve particle visibility and measurement accuracy in high-speed flow experiments. Through modifications of the CMOS circuit, pixel-level exposure and phase control mechanisms are introduced [6], enabling individualized exposure times and phase offsets per pixel within a single frame. This approach circumvents motion artifacts inherent in multi-frame synthesis High Dynamic Range (HDR) methods, although such multiple-exposure optimization provides limited improvement in the dynamic range of the high-speed camera.

Due to the limited dynamic range improvement of frame-based CMOS, two main methods are employed in the PIV systems to address the problem of overexposure caused by high-reflectance interfaces: the fluorescent particle method and polarization filtering technology.

The fluorescent particle method employs tracers that emit light at a longer wavelength (Stokes shift) when illuminated by the laser sheet. A bandpass filter is then used to block the specular reflection while transmitting the fluorescent signal, thereby significantly improving the signal-to-noise ratio near the interface [7]. Although effective for flow measurement, this approach has critical limitations: the inherent weakness of the fluorescent signal often results in an insufficient signal-to-noise ratio for high-speed flows, making precise interface identification challenging. Furthermore, the limited variety of fluorescent particles restricts their application in specialized flow fields.

The polarization method suppresses glare by exploiting the difference in polarization states between the specularly reflected light and the diffusely scattered light from particles. An analyzer placed in front of the camera is oriented to block the polarized reflection [2]. Its major drawback is the strong dependence on the angle of incidence, making it effective only near the Brewster angle, and thus unsuitable for curved or dynamically deforming interfaces. It also attenuates a portion of the useful particle scattering signal.

Therefore, while both methods can mitigate reflective glare to some extent, they are fundamentally limited by low signal strength and angular sensitivity, respectively. Consequently, neither technique can reliably achieve a simultaneous and precise measurement of the surrounding velocity field under complex interfacial conditions.

The core of estimating flow fields in overexposed areas of the gas–liquid interface is the problem of detecting and tracking the motion of weak signal particles in a strong light background. This requires a high-speed camera with a wide dynamic range to record the motion information of weak signals, as well as a corresponding low signal-to-noise ratio motion target processing algorithm. The primary advantage of utilizing dedicated cameras and data processing algorithms for particle image velocimetry in overexposed scenes is the enhanced particle detection capability in such scenes. At the same time, this solution is compatible with existing particles and optical devices in PIV experiments and has a wide range of applicability.

### 1.2. Neuromorphic Vision Sensor

The neuromorphic vision sensor (NVS), which includes the event camera and the spike camera, exhibits superior temporal resolution and a wide dynamic range. Pioneering studies have demonstrated their potential for particle image velocimetry.

#### 1.2.1. Event Camera

Event cameras, also known as dynamic vision sensor (DVS) or event vision sensor (EVS), asynchronously detect changes in light intensity to trigger event output [8], featuring microsecond-scale temporal resolution and a high dynamic range (120 dB, 1,000,000:1) [9]. This unique architecture provides exceptional sensitivity to dynamic targets [10] by responding exclusively to changes in light intensity of moving objects while ignoring static backgrounds, allowing for early prediction of the trajectory of high-speed objects. There have been related studies on event cameras in particle image velocimetry. Wang et al. [11] pioneered Stereo Event-PIV, the first demonstration of 3D flow field reconstruction using event vision. Their dual-camera system integrated sparse reconstruction and synchronization protocols to recover volumetric flow dynamics.

Recent advances in event-based imaging have introduced new paradigms for velocimetry and flow diagnostics. Event-based PIV enables high-speed flow measurements with superior temporal resolution [12], while event-driven 3D PTV provides direct access to dynamic wall shear stress [13]. The applications extend to turbulence studies, where reduced-order coordinates are efficiently extracted [14], and the ensemble PTV yields single-pixel turbulence statistics [15]. Benchmarking efforts validate the reliability of event-based methods using controlled experiments [16]. Moreover, the fusion of frame-based and event-based cameras has enabled super-time-resolution tracking [17], temporally adaptive PTV [18], and schlieren imaging [19]. Broader applications include atomization studies [20] and imaging in diffusive media [21].

Event-based cameras extend the dynamic range and suppress saturation, significantly improving the measurement accuracy near interfaces. However, several challenges remain unresolved, including reduced particle reconstruction accuracy in regions with complex refraction and local signal loss under strong scattering or multi-reflection conditions, which restrict their applicability under extreme experimental conditions.

#### 1.2.2. Spike Camera

The spike camera was proposed by Huang et al. [22] at Peking University. Its principle can be summarized as follows: each pixel independently integrates the photoelectric signal, and when the preset threshold is reached, it triggers a reset and outputs a spike. This spike sequence contains high-accuracy encoding information on pixel illumination changes, which can reconstruct images at any time. There are two methods for image reconstruction in spike cameras: (1) the intensity of light is inversely proportional to the spike interval; (2) the intensity of light is directly proportional to the number of spikes. Therefore, utilizing the spike interval (Texture from Inter-Symbol Interval (TFI)) or simply calculating the number of spikes over a period of time (Texture from Playback (TFP)), the complete image reconstruction of the intensity of light in the scenario can be obtained. The TFI method is suitable for clear imaging of moving scenarios, achieving clear imaging of fast-moving targets through the calculation of instantaneous light intensity. However, there is insufficient energy accumulation for slow-moving targets, and it is suitable only for a fast preview. The TFP method is suitable for clear imaging of static scenarios, similar to frame-based high-speed cameras with fixed exposure times, but still suffers from overexposure in reconstructed images of high illumination areas. Therefore, studying image reconstruction algorithms for spike cameras based on a specific speed is an important development direction for spike cameras.

Spike cameras have been preliminarily validated in tasks such as high-speed wind tunnel flow field observation and high-speed target recognition and tracking. Their sparse event-driven data structure provides a physical basis for constructing new visual perception algorithms, which can estimate optical flow based on continuous spike data streams [23]. Recently, Zhang et al. [24] used spike cameras for particle image velocimetry simulation and proposed the Spike Imaging Velocimetry (SIV) framework, an end-to-end deep learning model specifically designed for flow estimation in spike data. However, spike cameras have not yet been used to solve practical experimental problems in particle image velocimetry, especially in overexposed scenarios under complex interfacial conditions.

### 1.3. The Current Unresolved Difficulties in Overexposed Scenes

Although the above methods have been used to mitigate these overexposure effects, unresolved challenges remain, including strong scattering, multiple reflections, and signal loss in complex flow environments. Research and development are still needed for particle image velocimetry in overexposure scenarios.

- Frame-based high-speed cameras have insufficient dynamic range due to all pixels having the same integration time (high-speed cameras typically <60 dB, 1000:1), making it difficult to simultaneously capture high brightness and low brightness scenarios, as well as overexposed and non-overexposed regions;
- Event cameras are challenging to accurately depict moving particles in overexposed areas due to differential principles, as they lack sensitivity to signals of low-light intensity changes [25,26,27], such as slow motion, static backgrounds, and low-light intensity changes.

Spike cameras differ from frame-based cameras in that all pixels have a unified fixed exposure time imaging principle, and also differ from the differential principle of imitating frog and snake eyes in event cameras; spike cameras draw inspiration from the integration principle of the central fovea of the human retina. spike cameras fully record the photoelectric conversion process, retain complete details, and form spike data that can be processed into images and events. This gives the spike cameras a potential advantage in particle image velocimetry, particularly in complex lighting scenarios.

### 1.4. The Algorithm Proposed in This Study

In response to the problem of overexposure of images from the liquid–gas interface, difficulty in detecting particles at the liquid–gas interface, and unmeasurable velocity fields in frame-based high-speed cameras in particle image velocimetry, this paper proposes a particle image velocimetry algorithm based on spike camera adaptive integration. By segmenting the high-frequency digital spike signals collected by the spike camera into target backgrounds, high illumination background areas are suppressed, and the overexposure area is effectively controlled. The preprocessed spike signals are adaptively integrated on the basis of the spike frequency characteristics of particles with different background illumination areas and motion speeds to form a high signal-to-noise ratio reconstructed particle image, thereby improving the accuracy in particle image velocimetry of the liquid–gas interface.

## 2. Materials and Methods

Frame-based image cameras are based on a unified frame rate and fixed exposure time to sample the scenario, though their inherent limitations lie in the difficulty of achieving both high dynamic range and high temporal resolution simultaneously. The spike camera, on the other hand, adopts a non-frame-based spike integration mechanism, where each pixel works independently, breaking through the above contradiction.

For particle image velocimetry in overexposed scenes caused by high illumination in the liquid–gas interface, it is necessary to study an adaptive integral particle image velocimetry algorithm for high illumination environments. The key point is how to improve the signal-to-noise ratio of particle images in high illumination areas; while the improvement of the signal-to-noise ratio of particle targets depends on the matching of camera exposure time and particle features. Frame-based cameras only allow for the setting of a fixed exposure time, making it difficult to customize adjustments based on the characteristics of different particles in different areas. At the same time, when facing high illumination backgrounds, an excessively long exposure time can lead to overexposure of the area and loss of particle targets, whereas an excessively short exposure time may lead to underexposure of the area and difficulty in quantifying particles.

**Figure 3:**
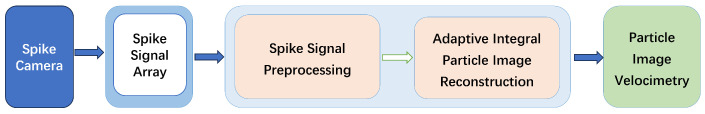
Structural diagram of the algorithm system.

This article focuses on the overexposure problem at the liquid–gas interface and the particle tracking problem in the overexposed region in particle image velocimetry applications using spike cameras. The algorithm system scheme designed includes three parts (as shown in Figure 3): signal acquisition based on spike cameras, spike signal preprocessing, and the adaptive integral spike image reconstruction algorithm.

The algorithm system comprises a Spike Signal Array module to collect high-frequency spike array signals, and a Spike Signal Preprocessing module to preprocess the spike array signals. The iterative integration strategy is used to distinguish between the background area and the target area, and an intensity coefficient based on saturation integration time is used to suppress high illumination background in the background area, effectively preventing overexposure; the Adaptive Integral Particle Image Reconstruction module performs adaptive integral imaging enhancement for particles of different velocities, adapting the optimal integration time for each particle to obtain the best imaging signal-to-noise ratio; the particle image velocimetry module calculates the velocity field of the reconstructed particle image.

### 2.1. Signal Acquisition Based on Spike Camera

**Figure 4:**
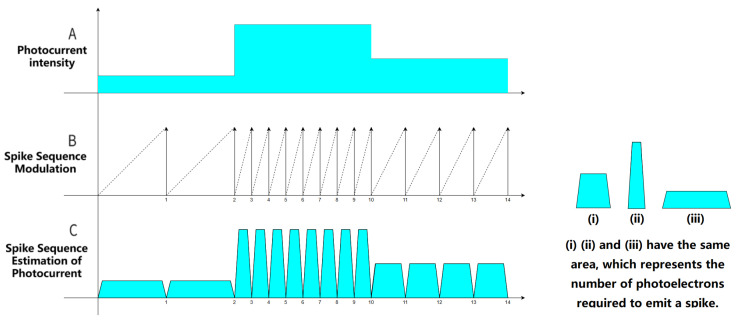
The principle of spike camera signal acquisition.

Unlike the conventional frame-based mode, where all pixels use the same fixed integration time, the spike-based mode is a per-pixel autonomous operation mode, where each pixel independently triggers a spike signal upon reaching an adjustable threshold. Through the calculation and processing of the digital spike signal, the accurate process of change in intensity of light is reflected and used for image reconstruction or perception of motion targets. The signal acquisition principle of the spike camera is shown in Figure 4.

The specific steps for signal acquisition of spike cameras are as follows:

- Each pixel independently receives photoelectrons and accumulates charges through integration;
- Charge accumulation causes the voltage to exceed the threshold, then emit a “1” spike and immediately reset, clearing the charge to zero;
- If the threshold is not reached within the output cycle, the emission will not be triggered and will remain at “0”;
- The continuous output of spike time series reflects the changes in intensity of light.

The formulas for triggering spike signals based on the accumulated instantaneous intensity of light and estimating the instantaneous intensity of light based on spike signals are as follows.

The triggering of spike signals is mainly determined by whether the accumulated instantaneous intensity of light reaches the threshold:

(1) $$\begin{array}{c} Spike_{t}=\left\{\begin{array}{cc} 0 & \tilde{I}\cdot \Delta t<\phi \\ 1 & \tilde{I}\cdot \Delta t>=\phi \end{array}\right. \end{array}$$

Among them, $Spike_{t}$ represents the value of the spike signal at time *t* in units of sampling time, $\tilde{I}$ is the instantaneous intensity of light, which is the constant intensity of light in a short period of time, Δt is the interval between time *t* and the time of the last spike in units of sampling time, and ϕ denotes the trigger threshold for the spike.

The trigger threshold ϕ of a spike camera determines the number of photoelectrons required to generate a spike. The threshold can usually be adjusted from a few hundred photoelectrons to tens of thousands of photoelectrons, so it should be dynamically adjusted according to the scene’s light intensity. The stronger the scene light, the higher the trigger threshold setting; the weaker the scene light, the lower the trigger threshold can be set.

An estimation of the instantaneous intensity of light can be achieved by calculating the time between two neighboring spikes (i.e., one interspike interval):

(2) $$\begin{array}{c} P_{t_{i}}=\frac{C}{\Delta t_{i}} \end{array}$$

Among them, $P_{t_{i}}$ represents the estimated value of the instantaneous intensity of light in pixels at time $t_{i}$ , *C* refers to the maximum dynamic range of the intensity of light, and $\Delta t_{i}$ represents the interspike interval corresponding to moment $t_{i}$ .

### 2.2. Spike Array Signal Preprocessing

For the overexposure scenario of high illumination at the liquid–gas interface, it is necessary to analyze the intensity of light in different areas and suppress the background in the high illumination regions.

We used a spike camera to fully capture the intensity of light. The higher the light intensity, the shorter the time interval required to reach the spike emission threshold; in contrast, and vice versa. Therefore, light intensity estimation can be performed for each background region based on the time interval of spike emission.

Considering the impact of noise and circuit on spike intervals, we will consider using spike intervals that are close in both the time and space domains to improve the accuracy of light intensity estimation.

Spike cameras estimate instantaneous light intensity through spike intervals, so by setting the integration time, it is possible to achieve cumulative imaging of instantaneous light intensity within the integration time similar to the frame-based camera.

**Figure 5:**
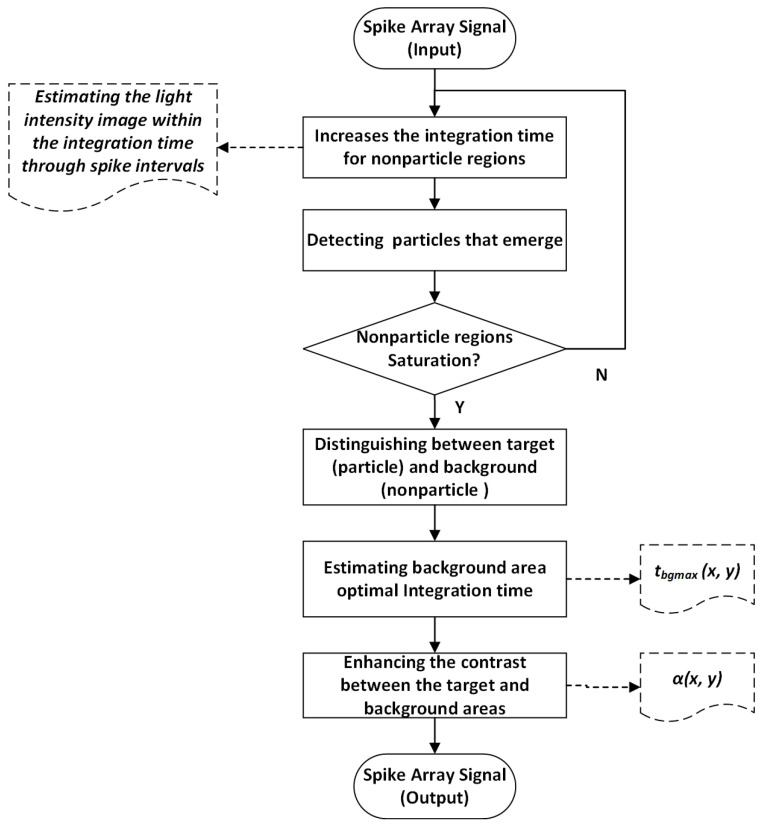
Spike signal array enhancement preprocessing.

The schematic diagram of the algorithm is shown in Figure 5.

Specifically, the system first performs a short integration time on the entire frame-based on the raw spike array data to prevent motion blur of high-speed particles and overexposure of background areas caused by long integration. At first, because of short integration, there were no particles in the entire region. The system then progressively increases the integration time for nonparticle regions, enabling detection of particles that subsequently emerge. It delimits new particle regions accordingly on the basis of these newly identified particles. After repeated iterations of the above process until all nonparticle regions reach saturation, it is considered that the particles that can be detected and resolved theoretically by the high-speed spike camera have been discovered, forming a fine division of all particle regions. Finally, the spike array data are divided into two categories: target area (particle regions) and background area (nonparticle regions).

At the same time, the integration time $t_{bgmax}\left(x,y\right)$ required for each pixel in all background areas (nonparticle regions) to reach saturation is calculated.

Based on the saturation integration time $t_{bgmax}\left(x,y\right)$ obtained above for each background region, the intensity of each background area can be determined. The smaller $t_{bgmax}\left(x,y\right)$ , the greater the intensity of the background area, while the larger $t_{bgmax}\left(x,y\right)$ , the smaller the intensity of the background area. Based on this inverse relationship, the strength coefficient Equation (3) is obtained as follows:

(3) $$\begin{array}{c} \alpha \left(x,y\right)=\frac{\beta }{t_{bgmax}\left(x,y\right)} \end{array}$$

Among them, α(x,y) represents the intensity coefficient of each pixel in the background region, β is the dimensionless weight for the suppression of background overexposure.

The background suppression coefficient β depends mainly on the light intensity of the brightest area in the background, which is used to balance the large dynamic background and provide a better image for subsequent particle tracking. In theory, the larger the background suppression coefficient β , the stronger the imaging ability for suppressing background light and achieving overall brightness balance. However, this will cause significant gradient segmentation at the edges of the particle and background regions, which will affect the overall imaging effect. Therefore, after multiple simulations and verification under current experimental conditions of strong laser illumination, setting the background suppression coefficient to 10 is a reasonable and feasible parameter.

For background area imaging, each background area pixel is subjected to the shortest spike sampling time to accumulate quantized grayscale values and divide them by the intensity coefficient α(x,y) , which can suppress the saturation of high-energy background areas, prevent overexposure, and ensure uniform illumination of all backgrounds.

Finally, in order to improve the computational accuracy of the flow field, contrast enhancement is performed on the target area where the particles are located.

### 2.3. Adaptive Integral Spike Image Reconstruction Algorithm

In addition, due to the insufficient integration time in overexposed regions with high illumination, the imaging signal-to-noise ratio of the particles is extremely low, making it difficult to be used for subsequent PIV velocity measurement. Therefore, it is necessary to enhance the particle signals in areas with high illumination overexposure. Considering the differences in energy intensity and motion speed between particles, it is necessary to allocate differential integration time for each particle based on its motion characteristics to achieve high-precision tracking and high signal-to-noise ratio imaging.

Using the signal-to-noise ratio (SNR) as the quality evaluation index for particle imaging. The SNR model is used primarily to describe the ratio between the target energy and the background noise. In particle imaging, this model reflects the contrast difference between the target and the background, which is applied for the detection and tracking of PIV particles. First, we assume that the expected light intensity of the target and background are $S_{t}$ and $S_{b}$ , respectively, which can be equivalently regarded as the total number of electrons arriving at a single pixel of the camera detector during the detection time. Due to the Poisson distribution effect in light propagation, the variance of the light intensity equals its expected value. Thus, the standard deviation of the intensity of the background light is $\sqrt{S_{b}}$ , representing the background noise. The SNR is the relationship between the expected value of the target light intensity and the standard deviation of the background light intensity, that is, $\frac{S_{t}}{\sqrt{S_{b}}}$ .

However, considering that particles are in a high-speed motion state and their individual pixel dwell time is limited, the actual theoretical Equation (4) is as follows:

(4) $$\begin{array}{c} \mathrm{SNR}\left(t\right)=\frac{S_{t}}{\sqrt{S_{b}}}=\left\{\begin{array}{cc} \frac{{S^{\prime }}_{t}\times t}{\sqrt{{S^{\prime }}_{b}\times t}}=\frac{{S^{\prime }}_{t}}{\sqrt{{S^{\prime }}_{b}}}\times t^{\frac{1}{2}} & t\leq t_{s}\\ \frac{{S^{\prime }}_{t}\times t_{s}}{\sqrt{{S^{\prime }}_{b}\times t}}=\frac{{S^{\prime }}_{t}\times t_{s}}{\sqrt{{S^{\prime }}_{b}}}\times t^{-\frac{1}{2}} & t>t_{s} \end{array}\right. \end{array}$$

Among them, $S_{t}$ and $S_{b}$ represent the total number of electrons reached by the particle and background signals, ${S^{\prime }}_{t}$ and ${S^{\prime }}_{b}$ represent the number of electrons reached by the particle and background signals per unit of sampling time, *t* represents the integration time, $t_{s}$ represents the individual pixel dwell time of the particle, and SNR(t) represents the target signal-to-noise ratio of the particle within the integration time *t*.

It can be observed that due to the constants of $S_{t}$ , $S_{b}$ , and $t_{s}$ , when the integration time is less than the individual pixel dwell time of the particles, the overall signal-to-noise ratio improves with increasing integration time; when the integration time is greater than the individual pixel dwell time of the particles, high-speed particles produce imaging blur and tailing due to displacement, resulting in an overall decrease in the signal-to-noise ratio with increasing integration time. Therefore, when the integration time is equal to the individual pixel dwell time, the signal-to-noise ratio of the particle target reaches its maximum, achieving the best particle tracking performance. Therefore, adopting spike camera adaptive integration based on the different intensity and velocity characteristics of particles can significantly improve the signal-to-noise ratio (SNR) of particles, which is more conducive to subsequent particle tracking and velocity measurement tasks.

Based on the original spike array data, the segmented particle regions are continuously integrated and the particle SNR at each integration time is calculated to obtain the relationship curve between the particle SNR of each particle region and the integration time. According to the previous conclusion, when the integration time is equal to the individual pixel dwell time, the particle SNR reaches its maximum value. Therefore, based on the change in the curve, the individual dwell time of the particle pixel can be determined, and the optimal integration time $t_{tg}\left(x,y\right)$ for all regions of the particles can be calculated.

Based on the optimal integration time $t_{tg}\left(x,y\right)$ for each particle region obtained above, the following Equation (5) is used for the adaptive integration of the particle regions:

(5) $$\begin{array}{c} t\left(x,y\right)=t_{tg}\left(x,y\right) \end{array}$$

**Figure 6:**
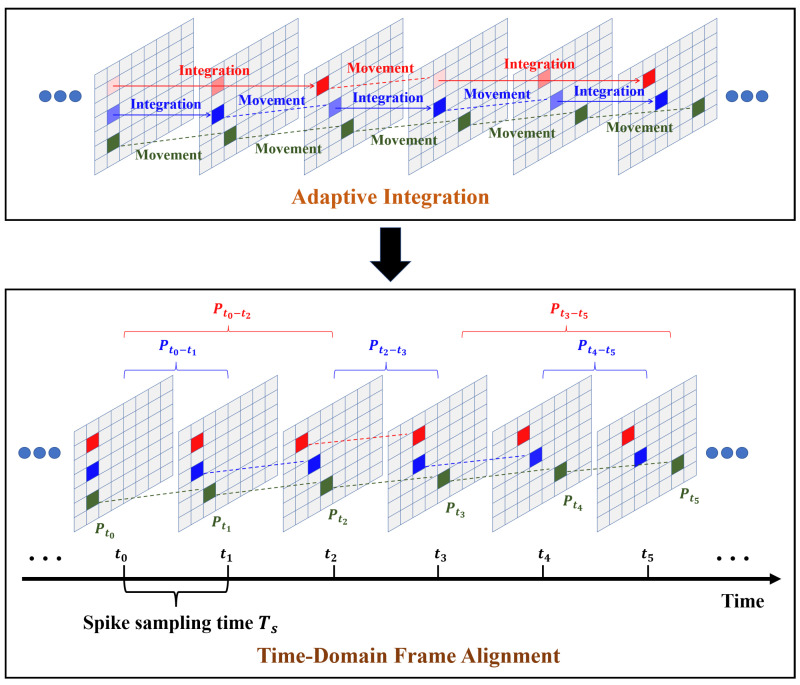
Particle regions adaptive integration method.

In the above equation, t(x,y) represents the integration time used by the pixels in each region of the particles. At this point, the integration time adapted to the individual pixel dwell time can be applied to each particle region to achieve the optimal target signal-to-noise ratio. As shown in the upper part of Figure 6, the optimal signal-to-noise ratio of particle tracking imaging is achieved by adapting the integration time of each particle region to its individual dwell time of pixels.

In addition, it is necessary to perform time-domain frame alignment on pixels with different integration intervals during the spike-array data imaging process. Using the spike sampling time as the time unit, the integration time of each pixel must be a multiple of the shortest spike sampling time.

As shown in the lower part of Figure 6, assuming that the initial sample moment of the particle target image in a certain area is $T_{m}$ , the spike sampling time is $t_{s}$ and the individual pixel dwell time of the particle target in a certain area is $t_{z}$ , $n=t_{z}/t_{s}$ samplings are required to achieve the best signal-to-noise ratio imaging effect for the particle. The integration time range is from $T_{m}$ to $T_{m+n}$ . At this time, the result of the integrated pixel value $P_{T_{m}-T_{m+n}}$ is obtained. After time-domain frame alignment, the corresponding pixel values in the imaging results of *n* samples from $T_{m}$ to $T_{m+n}$ are equal to $P_{T_{m}-T_{m+n}}$ . For the particle regions, since the integration time is the individual pixel dwell time, it means that the particles will not shift within this integration range. Therefore, using the frame replication method will not affect the actual position of the particles and can obtain imaging results that are adapted to the optimal integration time for all particles in any frame. The special case is illustrated by the green target in the lower part of Figure 6. When the dwell time is less than or equal to the spike sampling time, the energy integrated for this target is just the energy of a single sampling $P_{T_{m}}$ , and no integration is required.

**Figure 7:**
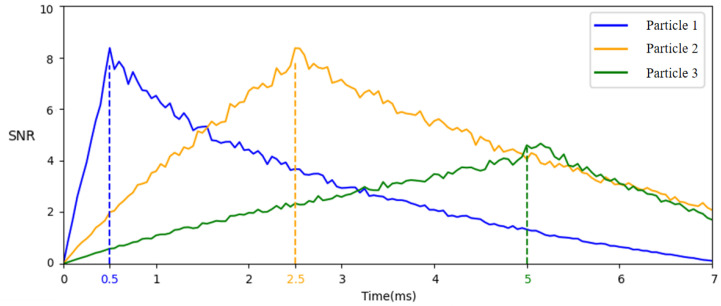
Theoretical curves of SNR over time for three types of typical particles. The calculation formula for SNR is shown in Equation (4).

To verify the effectiveness of the above algorithm, an individual-pixel SNR analysis is conducted on particles with three different motion speeds (Particle 1, Particle 2, and Particle 3) as a function of the integration time. The curve graph is shown in Figure 7. Based on the curve changes, the individual pixel dwell time of the three particles can be determined, with optimal integration times of 0.5, 2.5, and 5 ms, respectively.

**Figure 8:**
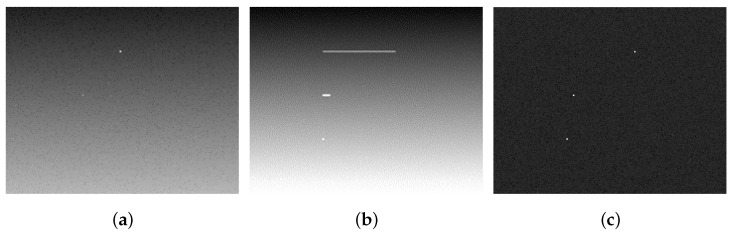
Particle imaging simulation under two working modes of frame-based cameras and adaptive integration of spike-based cameras: (**a**) Short exposure of the frame-based camera (1 ms, low-speed particle underexposure). (**b**) Long exposure of the frame-based camera (20 ms, high-speed particle dragging and overexposure). (**c**) Adaptive integration of the spike-based camera (no dragging and no overexposure).

Based on the optimal integration time of each particle, the proposed spike camera adaptive integration algorithm is used to compare the imaging effects with frame-based cameras at different exposure times, as shown in Figure 8. The three types of particle features from top to bottom in the scenario are fast-speed particles, medium-speed particles, and low-speed particles, respectively. The background illumination gradually increases from bottom to top in the scenario.

As shown in Figure 8, it can be seen that for frame-based camera imaging results, the best detection capability for Particle 1 can be achieved in a 1 ms short exposure mode, allowing for clear visibility of the particle. However, due to the short exposure time, it is difficult to accumulate sufficient energy for quantification imaging of Particles 2 and 3, which are weaker in intensity, resulting in a low signal-to-noise ratio of the targets; in the 20 ms long exposure mode, Particles 1 and 2, which are fast-moving particles, exhibit a tailing phenomenon. At the same time, overexposure occurs during long exposure, resulting in a low signal-to-noise ratio for Particle 3 approaching this region. Therefore, the fixed exposure time mechanism of frame-based cameras is difficult to accommodate multiple particles with different motion speeds in the same PIV scenario, and there is an overexposure problem caused by long integration in high illumination background areas, which ultimately makes it difficult to achieve optimal imaging effects for all particles for subsequent tracking and speed measurement.

For the imaging results obtained using the spike adaptive integration method, first, spike signal preprocessing is employed to distinguish particles from the background and suppress the background’s influence on particle imaging. At the same time, based on the independent and continuous integration characteristics of each pixel of the spike camera, different integration times can be set for particles with different background areas (high illumination areas, non-high illumination areas) and different velocities in the same PIV scenario, achieving optimal imaging capabilities for particle targets with various velocities in different background areas. Meanwhile, the high-frequency spike image reconstruction of the spike camera does not produce tailing or overexposure, preserving better time-domain features, which is beneficial for subsequent particle tracking and velocity measurement tasks.

## 3. Results

The proposed algorithm was validated using a two-pronged approach: (i) quantitative evaluation using synthetic particle images with a known ground-truth velocity field, and (ii) performance evaluation using experimental images captured from a physical flow with high illumination boundary conditions. The verification of the algorithm test is carried out through two methods: simulation and experiment.

### 3.1. Validation with Synthetic Data

#### 3.1.1. Generation of Synthetic Images

1. Synthetic particle image sequences were generated

Synthetic particle image sequences were generated based on the typical methodology for testing PIV cross-correlation algorithms [1]. This process involves three main steps: (i) defining a ground-truth velocity field, (ii) generating particle images by advecting particles according to this field, and (iii) superimposing a high-intensity region to simulate overexposure.

A 6400 × 6400 pixel laminar flow field was defined. The primary flow direction is horizontal, from left to right, over a stationary boundary located at the bottom of the domain. In line with the methods of [28], we simulate flow fields characterized by linear, quadratic, and logarithmic velocity gradients. This approach enables the evaluation of measurement techniques across a range of fundamental flow configurations. The main parameters of the simulated velocity field are a velocity range of 0.1–0.5 m/s, a particle diameter of 4 to 6 pixels, and a particle concentration of 4 pixels/ 32×32 area.

**Figure 9:**
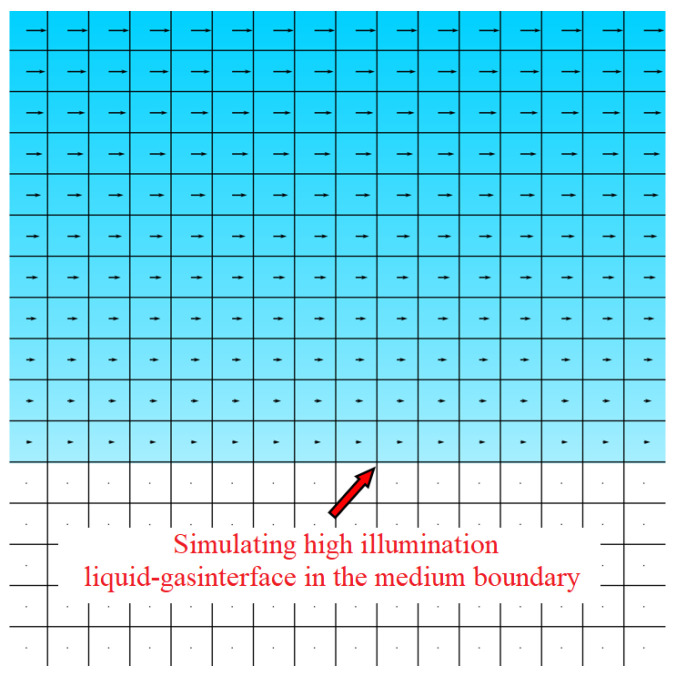
Schematic diagram of velocity field simulation, simulating high illumination liquid–gas interface in the medium boundary.

The velocity profile exhibits a classic liquid–gas interface structure: the velocity is zero at the stationary boundary and increases with the vertical distance from it. To simulate the challenging conditions, we superimposed a region of high illumination onto this liquid–gas interface. The resulting ground-truth velocity field is illustrated in Figure 9.

Particle Image Generation. The particles were initially seeded onto the computational domain, and their subsequent positions were advected according to the predefined velocity field to generate a sequence of particle images. The particle distribution must satisfy two constraints simultaneously: (i) The minimum number of particles per grid. Divide the 6400 × 6400 canvas into 200 × 200 grids (each grid 32 × 32). Set the minimum number of particles (4 by default) to ensure that in the random distribution, the number of particles in each 32 × 32 grid is not less than the minimum number of particles. If there are still some grids that do not meet the minimum particle count requirement when reaching the preset total particle count, continue to randomly add new particles until all grids meet the conditions; (ii) The minimum distance between particles. To avoid overly dense particles, it is also necessary to ensure that the center distance between any two particles is not less than the minimum particle spacing (adjustable parameter, currently set to 15.0 pixels). The specific method is to randomly select points on the canvas each time a new particle is generated; Determine the distance between the point and each generated particle. If they are all greater than or equal to the minimum particle spacing, pass; otherwise, retry. To prevent a dead loop, when there are too many retries (or attempts have been made to fill all available positions) and no new particles can be dropped, stop generating and continue with the subsequent simulation steps.

Simulate a high illumination liquid–gas interface. Add a horizontal light strip at the boundary of the input particle image medium to simulate reflection at the edge of the medium, and simulate scattering above the boundary of the medium based on Gaussian attenuation. Finally, a simulated flow field scenario with a high illumination boundary is obtained.

The simulation method utilizes the ray-tracing technique proposed by Rajendran et al. [29] to generate PIV and background-oriented Schlieren (BOS) images in environments with spatially varying density and refractive index, effectively reproducing refraction and scattering distortions near phase interfaces. To specifically emulate strong glare and local saturation at the boundary, a Gaussian intensity band is superimposed along the interface. The intensity distribution of the band is modeled as follows:

(6) $$\begin{array}{c} I\left(d\right)=I_{0}\exp\left(-\frac{d^{2}}{2\sigma^{2}}\right) \end{array}$$

where $I_{0}$ is the peak brightness in the center of the boundary, *d* is the normal distance from the boundary, and σ controls the width of the bright band. This approach reproduces the scattering halo and saturation effects caused by laser illumination, providing a controlled benchmark for testing particle detection and velocity reconstruction algorithms under overexposure scenarios.

2. Generate a camera simulation data stream and calculate the velocity field results

**Figure 10:**
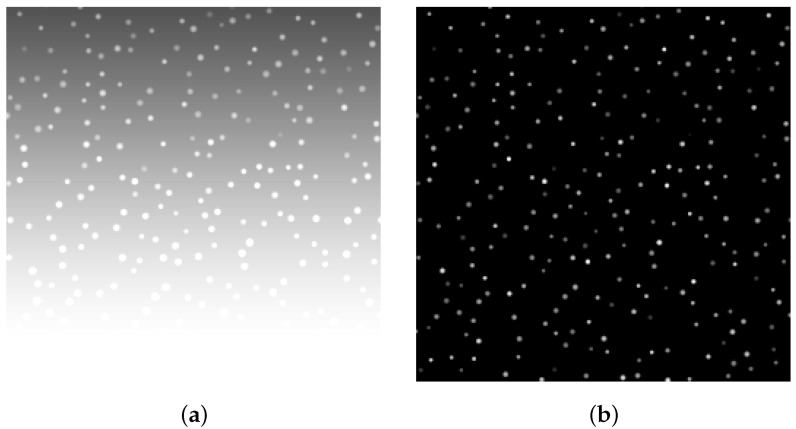
Comparison of simulation particle images: (**a**) Frame-based camera simulation. (**b**) Spike-based camera simulation.

Convert the simulated flow field scenario with high illumination boundary into frame-based camera images and spike data streams using frame-based camera models and spike-based camera models, respectively. Among them, preprocessing algorithms that can suppress background and adaptive integration algorithms based on particle motion characteristics are applied to spike data streams for particle image reconstruction. As shown in Figure 10, comparing the simulation imaging results of the two cameras reveals that our processing algorithm applied to the spike data stream can effectively mitigate the influence of high illumination areas in the liquid–gas interface on the overexposure of the particle image.

#### 3.1.2. Quantitative Analysis of Velocity Fields

**Figure 11:**
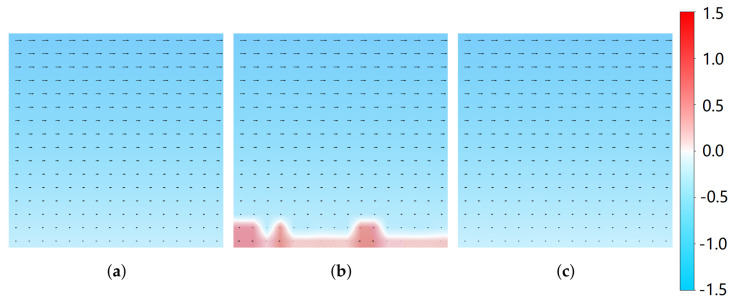
Comparison of velocity field calculation in simulation flow field: (**a**) The ground truth of the simulation flow field. (**b**) Frame-based camera. (**c**) Spike-based camera.

Using Pivlab 3.12.001 software (https://www.pivlab.de/), two frames with a maximum displacement of approximately 16 pixels are selected as a group, and the obtained frame-based camera velocity field and spike camera velocity field are compared with the true value of the flow field. Figure 11 qualitatively compares the results. The velocity field derived from the spike camera data demonstrates significantly higher accuracy in the high-intensity region, showing excellent agreement with the ground truth field. In contrast, the frame-based camera field exhibits large erroneous vectors in the overexposed region at the bottom (highlighted in red in Figure 11b).

**Figure 12:**
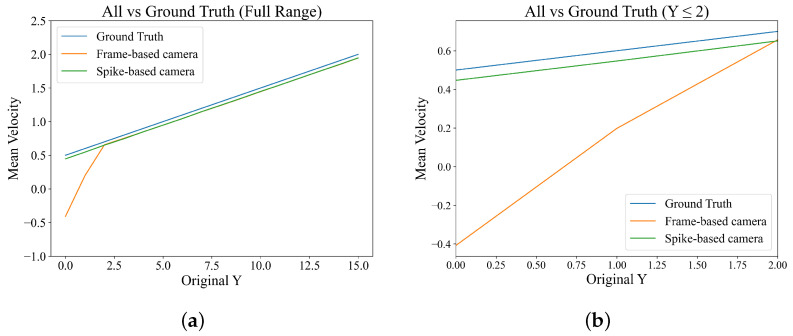
Comparison of velocity estimation errors in simulation flow field: (**a**) Comparison of errors across the entire area. (**b**) Comparison of errors in the high illumination areas.

The average velocity is calculated for each vertical axis and then validated against the true value of the simulation flow field, as shown in Figure 12.

We used the relative mean absolute error (RMAE) and root mean squared error (RMSE) to calculate the error of the algorithm in simulating the flow field.

The relative mean absolute error (RMAE) is normalized by the average magnitude of the observed values:

(7) $${\mathrm{RMAE}}_{\mathrm{velocity}}=\frac{\sum_{i=1}^{n}\left|v_{i}-{\hat{v}}_{i}\right|}{\sum_{i=1}^{n}\left|v_{i}\right|}$$

where *n* is the number of frames, $v_{i}$ is the actual value for the *i*-frame, ${\hat{v}}_{i}$ is the predicted value for the *i*-frame.

The root mean squared error (RMSE) for velocity is calculated as:

(8) $$\begin{array}{c} {\mathrm{RMSE}}_{\mathrm{velocity}}=\sqrt{\frac{1}{n}\sum_{i=1}^{n}{\left(v_{i}-{\hat{v}}_{i}\right)}^{2}} \end{array}$$

where *n* is the number of frames, $v_{i}$ is the ground truth velocity for the *i*-frame, ${\hat{v}}_{i}$ is the predicted velocity for the *i*-frame.

Calculate the error between the mean velocity of each longitudinal axis in each mode and the true value of the flow field, as shown in Table 1. Calculate the error between the mean velocity of each longitudinal axis and the true value of the flow field in the flow field results of the two cameras.

It is found that in the high illumination(overexposed) region at the bottom of the simulation flow field, the RMAE of the flow field results of the frame-based camera is 8.594 times that of the spike-based camera in conventional linear velocity gradient simulation scenarios. However, in the simulation of complex flow fields (quadratic linear and logarithmic velocity gradient) for velocity measurement, the advantage of the algorithm has decreased from the rate of the RSME, and further improvement is needed in the future.

In the non-high illumination (non-overexposed) region at the top of the simulation flow field, the errors of both have significantly decreased, with an RMAE ratio of nearly 1.009 times, as shown in Table 2, the effects of the two cameras are comparable, with the spike camera having a slight advantage.

The results of the simulation test have verified the effectiveness of the spike adaptive algorithm in calculating the velocity field in areas of high illumination, while achieving comparable results to those of a traditional frame-based camera in non-high illumination areas.

### 3.2. Experiment

#### 3.2.1. Experimental Setup

**Figure 13:**
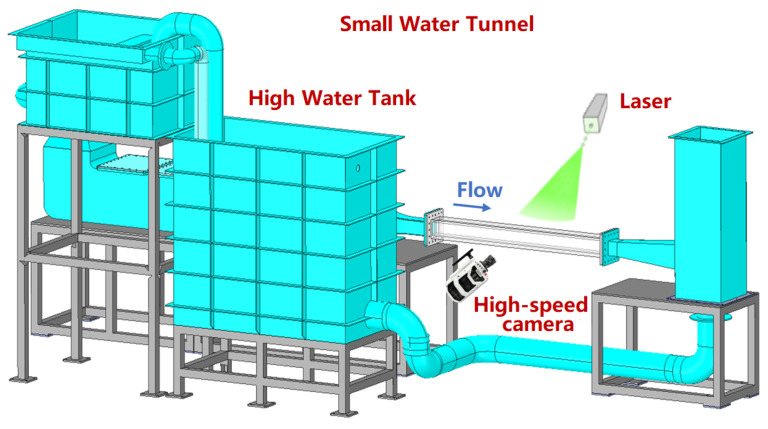
Equipment scheme design diagram.

The actual flow field observation experiment of this study is conducted in a customized gravity circulation small turbulent water tunnel, as shown in Figure 13. Essentially, it comprises a high-flow tank, a high-speed camera, a laser, and a section for testing incoming flow. The main technical indicators of the small water tunnel are shown in Table 3. The test section is made of high-transparency acrylic material to ensure optical accessibility, with a square cross-section of 100 mm (width) × 100 mm (height) and a total length of 1200 mm. A superhydrophobic coating is applied to the surface of the test sample and placed flat on the bottom wall, 800 mm downstream from the entrance of the test section. The coating is prepared using a commercial two-step spray method (NeverWet, USA), which allows a stable hydrophobic state. Under normal circumstances, the coating produces a specular reflection under laser illumination, resulting in overexposure of particle images around the coating, which makes it difficult to perform flow field calculations. For flow field visualization and subsequent PIV analysis, illumination is provided by a 30 W continuous-wave laser (LW-HP532-23L, Laserwave, China) with a working wavelength of 532 nm. The laser beam undergoes optical shaping through a combination of cylindrical and spherical lenses, forming a flat sheet that forms a laser sheet with a thickness of approximately 1 mm in the measurement area.

High-speed cameras are arranged in a direction perpendicular to the laser sheet light plane, and are used to record continuous sequences of particle images. This camera has a high frame rate (up to thousands of frames per second or more) and high spatial resolution (millions of pixels), which can capture images of rapidly moving particles clearly; by using the particle image velocimetry algorithm, particle displacement calculation and velocity vector field reconstruction calculation are performed to obtain the velocity distribution of the flow field.

The tracer particles used are polyamide resin tracer particles (Beijing Meicheng Technology Co., Ltd., Beijing, China) with an average diameter of 5 μ m. The imaging system comprised a 100 mm Tokina lens with an aperture set at f/2.8 . To address the magnification of the system, a camera calibration procedure was performed, which resulted in a determined spatial resolution of 1 pixel, corresponding to 0.02 mm in physical space.

In order to verify the effectiveness of the spike camera, the experiment mainly compared the Phantom (frame-based camera) and VidarPro (spike-based camera) commonly used in particle image velocimetry applications; two cameras use the same lens configuration, adjust the lens to fix the two cameras in the same field of view, and select the same effective pixel area ( 400×250 ) as the measurement window. The key technical parameters of the two cameras are shown in Table 4.

**Figure 14:**
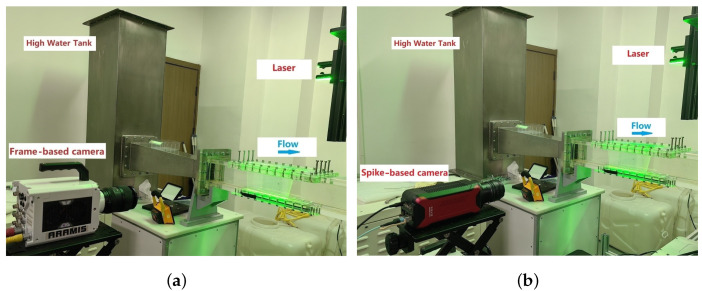
Experimental setup: (**a**) Frame-based camera. (**b**) Spike-based camera.

The experimental setup to compare the frame-based camera and the spike-based camera is shown in Figure 14.

#### 3.2.2. Accuracy Assessment of the Spike-Based PIV

Before conducting comparative experiments on the flow field of the superhydrophobic coating, it was essential to first validate the accuracy of the Spike-based PIV system. To this end, a benchmark validation experiment was conducted for a laminar flow. This experiment used a spike-based camera and the PIV algorithm to measure the laminar boundary layer at a mainstream velocity of *U* = 0.988 m/s.

Under these flow conditions, the velocity profile is expected to match the classical Blasius theoretical solution. For a direct comparison, the experimentally measured velocity component *u* was normalized by the mainstream velocity *U*, and the wall-normal coordinate *y* was normalized by the boundary layer thickness δ = 14.6 mm.

**Figure 15:**
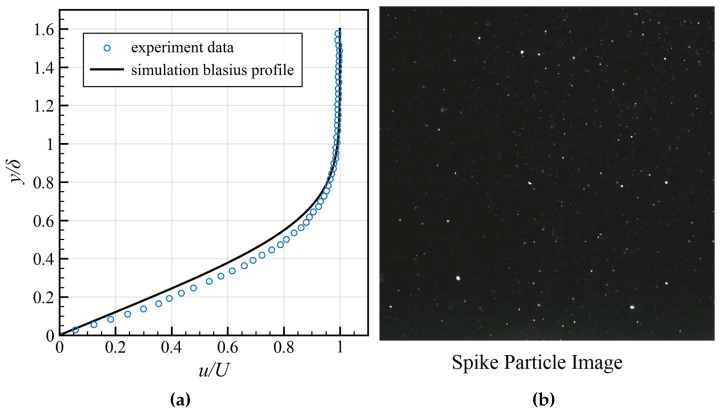
Comparison of the mean velocity profile measured by the spike-based camera against the Blasius profile: (**a**) Experiment results. (**b**) Spike particle image.

Figure 15 presents the comparison of system validation under laminar boundary conditions between the experimental data and the Blasius solution. As shown, the experimental data points obtained with the spike-based camera exhibit excellent agreement with the theoretical curve. This result validates the effectiveness of spile cameras in conventional flow field observations and confirms that our measurement system and analysis algorithm can accurately resolve the near-wall velocity field, providing a reliable baseline and confidence for subsequent slip measurements on high-reflection surfaces.

#### 3.2.3. Comparison of Frame-Based PIV and Spike-Based PIV

**Figure 16:**
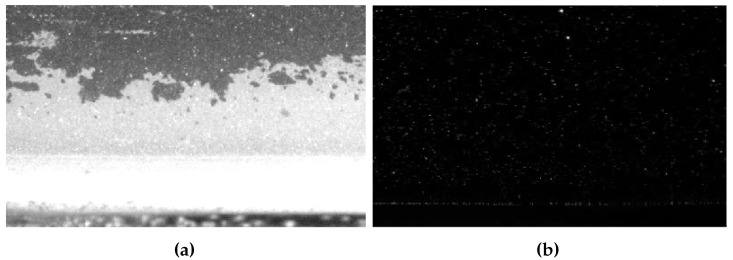
Particle image comparison: (**a**) Image from the frame-based camera, showing a large overexposed region. (**b**) Reconstructed image from the spike-based camera.

Comparison between frame-based PIV and spike-based PIV from two aspects: particle image and velocity field estimation. Figure 16 presents a direct comparison of the imaging performance at the boundary of the superhydrophobic coating under intense laser illumination. The image of the frame-based high-speed camera (Figure 16a) exhibits severe pixel saturation, creating a large overexposed region where the particles are completely obscured. In stark contrast, the image reconstructed from the spike camera data (Figure 16b) effectively mitigates this overexposure, revealing a high density of clearly resolved particles and the gas–liquid boundary within the same area.

**Figure 17:**
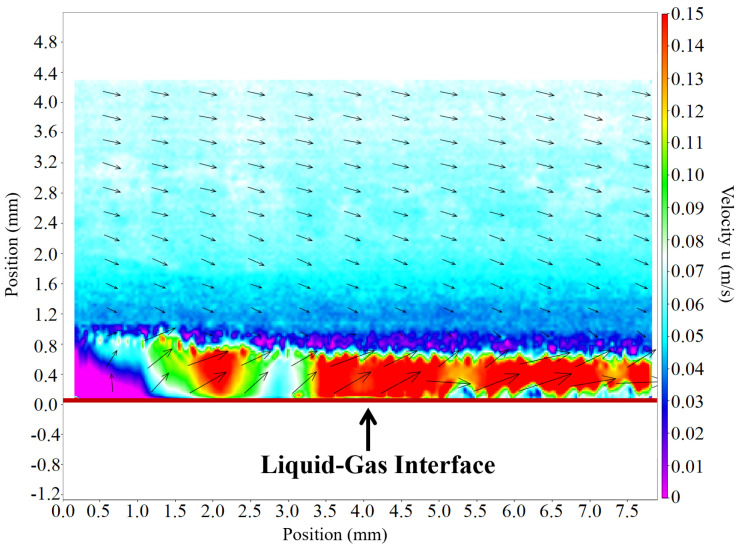
Comparison of PIV velocity fields: Frame-based camera: dense pseudo-vectors in the overexposed area, rendering the measurement invalid.

This marked difference in image quality directly impacts the accuracy of the resulting velocity fields, as illustrated in Figure 17. The lack of traceable particles in the overexposed image of the frame-based camera leads to a catastrophic failure in the PIV analysis, resulting in a field of spurious and erroneous vectors.

**Figure 18:**
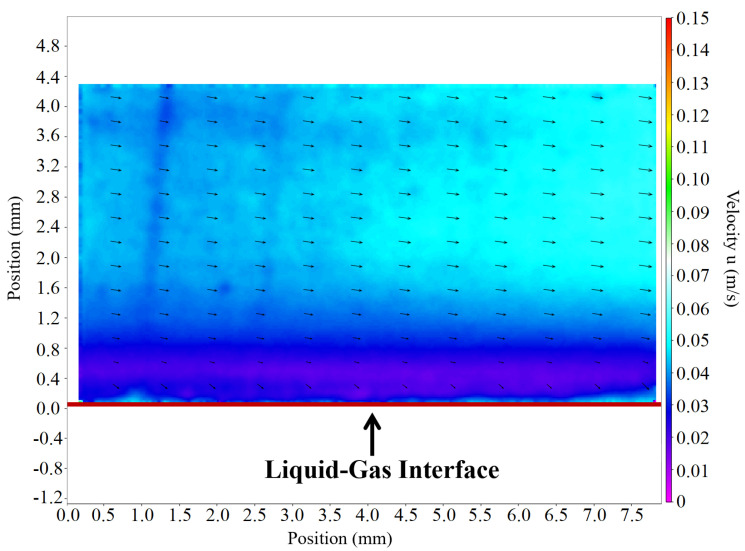
Comparison of PIV velocity fields: Spike-based camera: continuous and reliable velocity field throughout the area.

In contrast, the velocity field derived from the spike camera data is continuous and physically consistent throughout the measurement domain in Figure 18, including the challenging high-reflection boundary region. This is possible because the preserved particle information allows for reliable PIV correlation.

### 3.3. Discussion

The comparison results of the simulation flow field show that the velocity measurement error of the particle image velocimetry algorithm, based on the adaptive integration of the spike camera in the overexposed region at the boundary, is significantly lower than that of frame-based high-speed cameras and can form an accurate velocity field. In the experiment, a particle image velocimetry device is designed, through which the effects of frame-based cameras and spike-based cameras are compared. Spike cameras can effectively suppress the overexposure of the liquid–gas interface, improve the signal-to-noise ratio of particles in overexposed areas, and make the velocity field of the exposed area measurable.

Unlike existing deep learning-based spike camera velocity measurement methods [24], this algorithm employs a physics-driven adaptive integration strategy. Particle image velocimetry in overexposed scenes makes it difficult to obtain sufficient samples for training, learning, and validation due to the scarcity of data samples. This is why data-driven methods are challenging to apply in complex experimental scenarios, whereas the physics-driven adaptive integration method utilizes spike data that operate independently for each pixel and continuously utilizes high-frequency data for algorithm processing. Its core consists of preprocessing the high illumination area and the non-high illumination area by distinguishing particles from the background to suppress the high illumination background. At the same time, information on particles in the pixel residence time is accumulated to improve the particle signal-to-noise ratio, particularly to enhance particle recognition in high illumination areas, thereby achieving particle image velocimetry in these areas.

The flow field used to validate the algorithm system is a unidirectional flow scene. In the future, the algorithm system can be further optimized and extended to a flow field observation experiment of multiphase turbulent boundaries with multidirectional and complex illumination.

## 4. Conclusions

To address the challenges of overexposure of images under high illumination at the liquid–gas interface in particle image velocimetry applications, such as the low signal-to-noise ratio of particles in high illumination areas, the sparse identifiable particle numbers, and the consequent data loss in velocity fields, a particle image velocimetry method based on adaptive integration is proposed on the basis of a novel neural morphology spike camera. By preprocessing the high-frequency digital spike signals collected from the spike camera, the background and particles are distinguished, and the high illumination background is suppressed. The preprocessed spike signals are enhanced by an adaptive integration algorithm based on the motion characteristics of the particles, and then the flow field calculation is performed on the results. Quantitative evaluation of the algorithms is conducted through flow field simulation, and the effectiveness of the algorithm is further verified through an experiment on a liquid–gas interface flow under challenging high illumination conditions. The simulation and experiment results show that the particle image velocimetry algorithm based on spike camera adaptive integration can effectively mitigate high illumination overexposure in the liquid–gas interface, providing new ideas and solutions for particle image velocimetry in overexposure scenarios.

## Figures and Tables

**Table 1:** Comparison of velocity errors in high illumination areas.

Gradient of Speed Variation ^1^	Frame-Based Camera RMAE	Frame-Based Camera RMSE	Spike-Based Camera RMAE	Spike-Based Camera RMSE	RMAE Ratio ^2^	RMSE Ratio ^3^
Linear (CI HW)	0.723030 ± 0.112288	0.635536 ± 0.077221	0.084129 ± 0.002994	0.050836 ± 0.001823	8.594	12.502
Quadratic (CI HW)	0.404530 ± 0.075276	0.437883 ± 0.053206	0.027903 ± 0.001039	0.014044± 0.000363	14.498	31.179
Logarithm (CI HW)	0.205173 ± 0.029401	0.349597 ± 0.042329	0.113395 ± 0.003042	0.096370 ± 0.002478	1.809	3.628

^1^ CI HW: Half-width of the confidence interval, the values are reported as mean ± Half-width of the 95% confidence interval. CI HW, RMAE, and RMASE metrics are rounded to six decimal places. ^2^ RMAE ratio: Frame-based camera RMAE/Spike-based camera RMAE is rounded to three decimal places. ^3^ RMSE ratio: Frame-based camera RMSE/Spike-based camera RMSE is rounded to three decimal places.

**Table 2:** Comparison of velocity errors in non-high illumination areas.

Gradient of Speed Variation ^1^	Frame-Based Camera RMAE	Frame-Based Camera RMSE	Spike-Based Camera RMAE	Spike-Based Camera RMSE	RMAE Ratio ^2^	RMSE Ratio ^3^
Linear (CI HW)	0.038020 ± 0.000310	0.053413 ± 0.000448	0.037676 ± 0.000265	0.052953 ± 0.000370	1.009	1.009
Quadratic (CI HW)	0.058076 ± 0.000117	0.069745 ± 0.000123	0.057459 ±0.000243	0.069055 ± 0.000149	1.011	1.010
Logarithm (CI HW)	0.024865 ± 0.000365	0.042341 ± 0.000602	0.024367 ± 0.000299	0.041726 ± 0.000433	1.020	1.015

^1^ CI HW:Half-width of the confidence interval, the values are reported as mean ± Half-width of the 95% confidence interval. CI HW, RMAE, and RMASE metrics are rounded to six decimal places. ^2^ RMAE ratio: Frame-based camera RMAE/Spike-based camera RMAE is rounded to three decimal places. ^3^ RMSE ratio: Frame-based camera RMSE/Spike-based camera RMSE is rounded to three decimal places.

**Table 3:** Equipment introduction.

Parameter	Value
Overall Dimensions	Length 4.68 m × Width 1.1 m × Height 2.4 m
Flow Uniformity	0.5%
Velocity Range	0.1–1.9 m/s
Driving Method	Gravity-driven overflow type

**Table 4:** Comparison of experimental key technical indicators of high-speed cameras.

Camera type	Framerate	Resolution	Dynamic Range
VidarPro M1K20 ^1^	40,000 Hz	1000 × 1000	100 dB (100,000:1)
Phantom T2410 ^2^	10,000 Hz	1280 × 800	52 dB (400:1)

^1^ Vidar M1K20 camera max framerate 40,000 Hz, choosing the optimal framerate 20,000 Hz;the full frame resolution 1000×1000 , choosing the effective resolution 400×250 . ^2^ Phantom T2410 camera max framerate 10,000 Hz, choosing the optimal framerate 4000 Hz; the full frame resolution 1280×800 , choosing the effective resolution 400×250 .

## Data Availability

The raw data supporting the conclusions of this article will be made available by the authors on request.
